# Medication Errors at a Tertiary Hospital Intensive Care Unit

**DOI:** 10.7759/cureus.20374

**Published:** 2021-12-12

**Authors:** Abdullah E Laher, Callistus O Enyuma, Louis Gerber, Sean Buchanan, Ahmed Adam, Guy A Richards

**Affiliations:** 1 Emergency Medicine, University of the Witwatersrand, Johannesburg, ZAF; 2 Paediatrics, University of Calabar, Teaching Hospital, Calabar, NGA; 3 Urology, University of the Witwatersrand, Johannesburg, ZAF; 4 Critical Care, University of the Witwatersrand, Johannesburg, ZAF

**Keywords:** adverse drug reaction, drug administration, drug prescription, icu, medication errors

## Abstract

Background

The intensive care unit (ICU) generates more medication prescriptions per patient day than any other unit in the hospital. The dynamics of the ICU environment, coupled with the complexity of patient pathology, increases the risk of medication errors. This study aimed to evaluate the incidence and spectrum of medication errors in an adult general ICU in Johannesburg, South Africa.

Methods

A retrospective chart review was conducted at a 19-bed ICU in a tertiary-level hospital in Johannesburg. Data were independently collected by two of the study investigators. The doctors’ prescription and the nurses’ administration section of patient bedside charts were scrutinized for drug prescription and administration errors.

Results

Of the 656 patient days studied, 3237 drugs (5.6 drugs per patient day) were prescribed. There were a total of 359 medication errors, comprising 237 (66.0%) prescription and 122 (34.0%) administration errors. The total error rate per 1000 patient days was 621.1, while the total error rate per 1000 drug prescriptions was 110.9. The most common errors were incorrect dose prescribed (n=69, 19.2%), incorrect dosing interval prescribed (n=48, 13.4%), incorrect dose administered (n=42, 11.7%) and failure to administer the prescribed drug (n=38, 10.6%).

Conclusion

The overall occurrence of medication errors is high but is in keeping with general international trends. Targeted interventions should be implemented to minimize the frequency of medication errors in the ICU and consequent risk to patients.

## Introduction

Medication errors, which can be defined as any error in drug prescription, dispensing, and administration that may or may not lead to patient harm [1], are responsible for 78% of serious errors in the intensive care unit (ICU) [2]. The prevalence of medication errors in the adult ICU ranges from 1.2 to 947 errors per 1000 patient days [3]. The number of errors is important as these errors, and their associated adverse drug events (ADE), may significantly impact patient outcomes in the ICU [4].

In general, patients admitted to the ICU are prescribed twice as many medications as those admitted to the general wards and are therefore more susceptible to medication errors [5]. Other risk factors for errors in the ICU include; the severity and complexity of patient illness, unavailability of the patient’s chronic medication list, the need for sedation and mechanical ventilation, extremes of age, provider inexperience, inadequate supervision of junior staff, lack of communication between ICU staff, frequent staff changes, sleep deprivation of staff, high patient to nurse ratio, difficult working conditions, frequent changes in medications and doses, non-standardized dosing regimens, inaccurate patient weight estimation, the requirement of life-saving interventions, high number and complexity of medical interventions, prolonged length of ICU stay and the use of novel treatments [6-9].

The spectrum of medication errors is broad and includes prescription and administration errors related to incorrect dosing, incorrect frequency and route of administration, poor comprehension of the initial order, omission of a previously prescribed drug on the drug chart, failure to review the medication before transcription, mislabeling and incorrect drug reconstitution [10-13].

Although an earlier South African study evaluated medication errors among patients in a pediatric ICU, little is known about the incidence and type of medication errors that occur in an adult ICU in this region [14]. Therefore, we conducted this study to evaluate the incidence and spectrum of medication errors in an adult ICU at a tertiary-level hospital.

## Materials and methods

This was a retrospective cross-sectional study of the medical records (ICU charts) of patients that were admitted to the adult general ICU of a tertiary-level hospital situated in the Gauteng province of South Africa. The hospital has approximately 1000 beds in total, with six distinct ICU sections. The adult general ICU comprises 19 beds. Critical care specialists generally manage patients alongside critical care fellows in training and registrars from various specialties who rotate through the ICU for approximately three to four months as a requirement of their training. Medication prescriptions are handwritten/transcribed every morning onto the daily patient ICU charts by the registrar before discussion with the critical care fellow and critical care specialist. Each ICU chart spans a 24 hours starting, from 6 am until 6 am the following morning.

Permission to conduct the study was granted by the head of the ICU and the hospital manager, while ethics clearance was obtained from the Human Research Ethics Committee of the University of the Witwatersrand (Clearance certificate M170456). Before the initiation of data collection, the study investigators who were tasked with collecting data were trained by the study supervisors regarding the methods and principles of data abstraction from medical records. Data were independently collected by two study investigators from the daily patient ICU charts over the period 1^st^ April to 10^th^ May 2018. Drug prescription and administration sections of each chart were analyzed for errors. Findings were recorded in a specifically designed data collection sheet. The two study investigators thereafter compared their findings and resolved any conflicting information after mutual discussion and agreement among the study investigators. The process of data collection was periodically monitored by the study supervisors.

ICU charts with less than half a day (<12 hours) of recorded data (e.g., admission during the latter part of the day or discharge/death during the early part of the day) were excluded. The errors recorded were incorrect drug, incorrect dose, incorrect dosing interval, incorrect omission of drugs on the subsequent days’ charts, drug incorrectly prescribed after prior stop order, incorrect unit documented, prescription change not signed off, drug ordered verbally but not prescribed on the chart, an incorrect drug administered, incorrect dose administered, administration at incorrect dosing interval, a drug administered after a stop order on the chart, and prescribed drug not administered.

Data collected was subsequently entered into Microsoft® Excel® (Microsoft 365, Version 16.0.13029.20232) (Microsoft Corporation, Redmond, WA, USA) and thereafter subjected to statistical analysis. Frequency, percentage, error rate per 1000 patient days, and error rate per 1000 drug prescriptions were calculated and tabulated. The mean and standard deviation was calculated for the number of drugs prescribed and administered per patient day. Study reporting conformed with STROBE (strengthening the reporting of observational studies in epidemiology) guidelines [15].

## Results

There were a total of 656 ICU charts (patient days) over the study period, of which 78 were excluded due to either late admission time (n=37) or death/ICU discharge during the early hours of the day (n=41). Therefore, a total of 578 charts were included in the final analysis.

Overall, 3237 drugs were prescribed, giving an average of 5.60 (±2.72) drugs prescribed per patient day, while 3252 drugs were administered, giving an average of 5.63 (±2.88) drugs administered per patient day. There were a total of 359 medication errors, comprising 237 (66.0%) drug prescriptions and 122 (34.0%) drug administration errors. The total error rate per 1000 patient charts was 621.1, while the total error rate per 1000 prescriptions was 110.9. Table 1 describes the various types of drug prescription and drug administration errors that were identified, the error rate per 1000 patient days, and the error rate per 1000 drug prescriptions for each of the error types.

**Table 1:** The various types of drug prescription and transcription errors identified in the ICU charts analyzed *Incorrect administration that was not in keeping with the prescription, when the prescription was correct

Error Type	n (%)	Error rate per 1000 patient days	Error rate per 1000 drug prescriptions	
Drug prescription errors	237 (66.0)	361.3		73.2
Incorrect drug	21 (5.8)	36.3	6.5	
Incorrect dose	69 (19.2)	119.4	21.3	
Incorrect dosing interval	48 (13.4)	83.0	14.8	
Drug erroneously omitted on subsequent days charts	19 (5.3)	32.9	5.9	
Drug prescribed after prior stop order	22 (6.1)	38.1	6.8	
Incorrect SI (international system) unit documented	20 (5.6)	34.6	6.2	
Prescription changes not signed off	21 (5.8)	36.3	6.5	
Drug ordered verbally but not prescribed on chart	17 (4.7)	24.4	5.3	
Drug administration errors*	122 (34.0)	186.0		37.7
Incorrect drug	4 (1.1)	6.9	1.2	
Incorrect dose	42 (11.7)	72.7	13.0	
Incorrect dosing interval	27 (7.5)	46.7	8.3	
Drug administered after stop order on chart	11 (3.1)	19.0	3.4	
Prescribed drug not administered	38 (10.6)	65.7	11.7	
Total	359 (100)	621.1	110.9	

The most common errors were incorrect dose prescribed (n=69, 19.2%), incorrect dosing interval prescribed (n=48, 13.4%), incorrect dose administered (n=42, 11.7%) and prescribed drug not administered (n=38, 10.6%). The error rate per 1000 patient charts for each of these was 119.4, 83.0, 72.7, and 65.7, respectively, while the error rate per 1000 drug prescriptions was 21.3, 14.8, 13.0, and 11.7, respectively.

## Discussion

Although medication errors in the ICU have been well described internationally, to our knowledge, this study is the first to have investigated the incidence and spectrum of medication errors at an adult ICU in South Africa. Notable findings of this study are that 1) the overall drug prescription rate was 5.60 drugs per patient day, 2) prescription and administration errors accounted for 66% and 34% of the overall errors, respectively, 3) the overall error rate per 1000 patient days was 621.1 (62%), 4) the overall error rate per 1000 drug prescriptions was 110.9 (11%), and 5) incorrect dose prescribed (19.2%) and incorrect dosing interval prescribed (13.4%) were the most frequent error types.

Compared to the findings of this study, Macfie et al., in an integrative review that included 40 studies about medication errors in the ICU, reported a large variation in the incidence ranging from 5.1 to 967 errors per 1000 patient days. Although the incidence of ADE and its associated cost were not determined in our study, the authors reported that the incidence of ADE among the included studies ranged between 1 to 96.5 per 1000 patient days. Additional related costs ranged from $347 to $6647. Due to the wide variation in these figures as a result of the marked diversity between studies with regard to nomenclature, the quality of data collection, and study methodology, the authors were unable to subject their data to a meta-analysis [16]. Another study of a similar design which reviewed the findings of 20 studies, reported that the frequency of errors in adult ICUs ranged from 1.2 to 947 per 1000 patient days. The authors in this study attributed the large variation in error rates to the mechanisms of error detection, the number and type of ICUs evaluated, the existing prescription technology in the ICU, and the definitions of medication errors that were used [3].

A further study that was conducted at a medical ICU in Ethiopia reported that an average of 8.8 drugs were prescribed per patient. Of the 882 prescriptions, there were 359 (40.7%) errors identified, with at least one prescription error identified in 24.42% of the total number of medication prescriptions. Omission errors (42.89%), incorrect drug combinations (28.13%), incorrect abbreviations (13.37%), incorrect dosing (8.36%), incorrect frequency of administration (5.01%) and incorrect indication (2.23%) were the most common prescribing errors identified [17]. Another study that was conducted in Boston, USA, reported that there were 530 errors among the 10,070 medication orders (5.3%), with omission errors (53%), dosing errors (15%), incorrect frequency (8%), and incorrect route (5%) being the most frequent errors [18]. A separate study conducted in Denmark detected 1065 errors out of 2467 opportunities (43%). The most common error types were failure to order a drug and omission of a dose [19]. In a systematic review that included 45 studies across 10 middle eastern countries, prescription errors ranged from 7.1% to 90.5%, while administration errors ranged from 9.4% to 80%. The most common types of prescribing errors were incorrect dose and incorrect frequency [7]. Another study reported that omission errors accounted for 52% of transcription errors [20]. 

In the current study, there was a slight discrepancy between the overall number of prescribed (n=3237) and administered (n=3252) drugs, which may suggest either verbal prescription by the clinician without a written prescription or nursing staff-initiated drug administration following internal protocols. Besides the potential associated risk of medical litigation [21], failure to document administered medications may also lead to drug-drug interactions and other drug adverse events if the clinician forgets to account for these drugs during patient review [22]. Hence, nursing staff needs to ensure that doctors follow up a verbal order with a written prescription as soon as possible.

Since medication errors are a common but preventable cause of adverse medical events in the ICU and pose a significant threat to patient safety, a conscious effort is required to reduce the incidence of medication errors [3]. An integrated approach involving the entire healthcare team together with the incorporation of appropriate interventions is critical. Interventions that have been shown to reduce the incidence of medication errors include electronic prescribing, the use of smart pumps, barcoding of drugs, staff training, senior oversight, clinical pharmacist involvement, and mandatory review of drug prescriptions [16,23]. However, access to many of these interventions may be limited in low-resource settings. Future studies should aim to investigate the extent of medication errors in ICUs across South Africa and to determine the effect of various interventions, including the role of an in-house pharmacist [24], in reducing the incidence of medication errors locally.

There are some limitations to this study. First, this was a single-center study; hence our findings may not be generalizable to other settings. Second, the adverse event rate secondary to each of the errors was not determined. Third, since medication dosing in the ICU can be complex due to dynamic changes in patient physiology, renal function, and hepatic function, the extent of medication errors may have been under- or over-reported as a result of using non-standardized or off-label dosing regimens.

## Conclusions

The overall occurrence of medication errors in this study was high but in keeping with general international trends. Targeted interventions should be implemented to minimize the frequency of medication errors in the ICU and subsequent risk to patients.
